# The efficacy of repetitive transcranial magnetic stimulation (rTMS) for young individuals with high-level perceived stress: study protocol for a randomized sham-controlled trial

**DOI:** 10.1186/s13063-021-05308-3

**Published:** 2021-05-25

**Authors:** Jingsong Wu, Mengyu Han, Youze He, Xiaoting Xie, Jian Song, Xiujuan Geng

**Affiliations:** 1 Fujian University of Traditional Chinese Medicine, Fuzhou, China; 2 Shenzhen Research Institute, The Chinese University of Hong Kong, Hong Kong, China; 3 Brain and Mind Institute, The Chinese University of Hong Kong, 4F, Hui Yeung Shing Building, Shatin, N.T., Hong Kong, China

**Keywords:** Perceived stress, Repetitive transcranial magnetic stimulation, Young adult, Theta burst stimulation, Magnetic resonance imaging

## Abstract

**Background:**

High level of perceived stress may result in negative effects both psychologically and physically on individuals and may predispose onset of mental disorders such as depression, anxiety, and posttraumatic stress disorder. However, there is no suitable intervention for it. Repetitive transcranial magnetic stimulation (rTMS) studies have shown its therapeutic efficacy in treatment resistant patients with stress-related disorders. Here we describe an exploratory study protocol to investigate the effect of the intervention for the individuals with high level of stress.

**Method:**

This is a single blinded, randomized sham-controlled trial, targeting at young healthy adults aging from 18 to 24 years old. Forty eligible volunteers will be recruited and randomly divided into active and sham rTMS group. All subjects will take a set of neuropsychological and biological assessments and MRI scanning before and right after the intervention. During the interventional period, 12-session stimulations will be performed in 4 weeks with three sessions per week. The primary outcome will detect the difference of Chinese 14-item perceived stress scales between active and sham rTMS groups after intervention. Secondary outcomes will examine the differences of other affective measurements, level of cortisol, and MRI-derived neural functional measures between the two groups after intervention.

**Discussion:**

This trial aims to examine the effect of the 12-session rTMS intervention on individuals with high level of perceived stress. Positive or negative findings from any of the outcome measures would further our understanding of the efficacy of the stimulation and its neural impact. If effective, it would provide an evidence for a new treatment for high perceived stress.

**Trial registration:**

Chinese Clinical Trial Registry (ChiCTR1900027662). Registered on 23 November 2019. And all items of the WHO Trial Registry Data set can be found within the protocol.

**Supplementary Information:**

The online version contains supplementary material available at 10.1186/s13063-021-05308-3.

## Background

Stress has been called the “health epidemic of the 21st century” by the World Health Organization, which is a personalized phenomenon and varies among individuals depending on personal vulnerability and resilience [1]. Stress is a common problem in modern life with negative outcomes, especially among young adults including college students who may experience undue amount of stress related to the pressure to succeed [2–4]. High level of perceived stress may result in negative effects comprising both psychological and physical aspects on individuals, such as depression [5], decrease of immune functions, and increase of cardiac risk factors [6]. However, due to the absence of suitable interventions for stress, the method of treatment still remains marginal in clinical practice [7].

Repetitive transcranial magnetic stimulation (rTMS), one of the brain stimulations for conscious subjects, has recently gained emerging interest as a non-invasive intervention for a number of treatment-resistant psychiatric disorders [8, 9]. RTMS allows researchers to explore the properties and organizations of neural function and has the ability to drive the alterations of brain plasticity [7, 10]. It can induce the neuro-modulation and change the excitability of cerebral motor and prefrontal cortices by applying different stimulation frequency. Furthermore, the usage of rTMS has been approved by US Food and Drug Administration (FDA) in October 2008 and its efficacy to some stress-related affective disorders (e.g., depression, anxiety and post-traumatic stress disorder) has been supported and recommended in the most recent rTMS guideline [11, 12]. However, it is unknown whether rTMS can relieve the symptoms of perceived stress. Given the effect of rTMS to stress-related diseases and the absence of non-pharmacological intervention for reducing perceived stress, rTMS could be a potential intervention technique to investigate for the individuals with high level of perceived stress.

Previous studies have suggested that high level of perceived stress may cause alterations of cerebral morphological and functional plasticity and is associated with the structural and functional changes of prefrontal cortex (PFC) [13–15]. Moreover, a sequential process of hypothalamic-pituitary-adrenal (HPA) axis disturbance caused by high level of perceived stress may result in increase of the secretion of cortisol to a higher level [7, 16]. Meanwhile, evidence has shown that rTMS can decrease the density of cortisol level in saliva and blood [17–19], and the stimulation to dorsolateral prefrontal cortex can induce changes in the HPA axis and immune function in the form of cytokine production in individuals with depression and posttraumatic stress disorder [20]. Therefore, the current protocol is designed to rigorously test the efficacy of rTMS in high-level perceived stress, and its effects on brain plasticity and secretion of cortisol.

### Study aims

The primary aim is to test the hypothesis that rTMS stimulation may assist in relieving the stress perceived in daily life. Secondarily, we aim to (a) examine effects of rTMS on brain function related to the site of stimulation and (b) explore whether the level of cortisol could be useful in identifying the efficacy of rTMS, decreased after intervention.

## Methods/design

### Study design

**Fig. 1:**
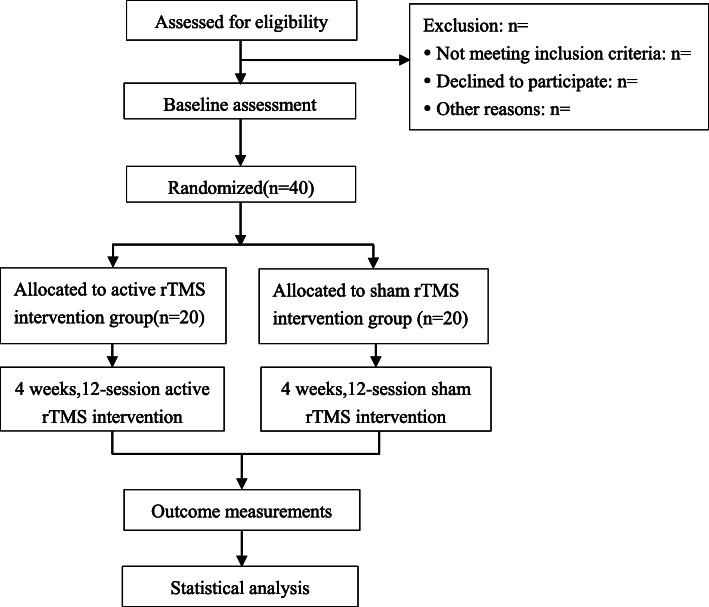
Flow chart of study design

**Fig. 2:**
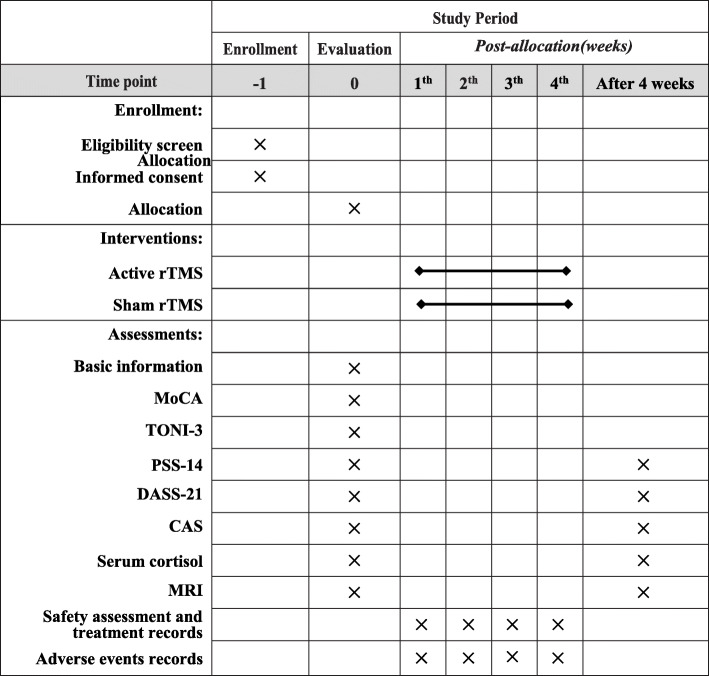
The schedule of enrollment, interventions, and assessments. Abbreviations: MoCA, Montreal Cognitive Assessment; TONI-3: Test of Nonverbal Intelligence, Third edition; PSS, Perceived Stress Scale; DASS, Depression, Anxiety and Stress Scale; CAS, Chinese Affective Scale; MRI, magnetic resonance imaging

This is a single blind, randomized controlled trial, targeting at young healthy college students. Forty high level of perceived stress subjects will be recruited and randomly (1:1) assigned to either (1) a 12-session treatment protocol of active rTMS or (2) a 12-session treatment protocol of sham rTMS. Participants will be evaluated for outcome measures before and after the intervention, in addition to the primary efficacy measure of the Chinese 14-item perceived stress scale (PSS-14), and secondary measures of other neuropsychological, clinical, and biological assessments and MRI scanning will be obtained. The executors of intervention will be trained by standard operation procedure following the protocol setting. In addition, the trial’s randomization and allocation will be conducted and supervised by an independent researcher. The protocol study follows the SPIRIT recommendations. For the SPIRIT checklist, see Additional file 1, and the study design is presented in Figs. 1 and 2.

### Participants

This trial is designed to enroll 40 healthy volunteers with the PSS-14 score of more than 25, and aging from 18 to 24 years old. All eligible participants will be recruited from universities and nearby communities, who are willing to join this project. Before entering, we will present a full explanation about the purpose of our trial, types of intervention, and the potential risks and benefits of rTMS. Besides, their willingness will be respected if they would not continue our trial for any reason.

### Enrollment

#### Inclusion criteria

All the participants should meet all following criteria: (1) ages are from 18 to 24, with no gender restriction; (2) scores of Perceived Stress Scale are over 25 [21]; (3) have a high level of educational experience (above university or at college); (4) clear consciousness; (5) no intracranial cerebrovascular disease; (6) no psychiatric disease; (7) no history of brain damage; (8) no epilepsy or family history of epilepsy; (9) no neurodegenerative disease; (10) no contraindications of MRI examination; (11) had not taken similar TMS experiment previously; and (12) sign the informed consent.

#### Exclusion criteria

Volunteers should be excluded if (1) cannot tolerate the MRI scan for any reasons; (2) cannot tolerate the TMS intervention; (3) having uncorrectable movements during the MRI scan; (4) unable to give informed consent; and (5) having implant devices, such as deep brain stimulator, cochlear prosthesis, cardiac pacemaker, etc., which are contraindicated to MRI or TMS.

#### Withdrawal or dropout criteria

Interventions in either active group or control group will be stopped if participants meet the following criteria: (1) subject is unwilling to continue the intervention for any reasons; (2) subject suffers from any serious adverse events during the intervention period (e.g., seizures); and (3) subject develop a serious disease (e.g., traumatic injury, stroke).

### Intervention

The intervention of rTMS will be carried out using Magstim Rapid^2^ stimulator (Magstim, Wales, UK) with a standard 70-mm figure-of-eight coil (Active Air film coil, Magstim, Wales, UK). All subjects included should be diagnosed by physicians or clinicians from neuro-rehabilitation or neurology department before entering the experiment to ensure whether they are suitable to participate in this study.

For the stimulation schedule, previous studies have evidenced that the stimulation with intermittent theta burst stimulation (iTBS) to left and continuous TBS (cTBS) to right DLPFC is a safe and feasible strategy to patients with major depression and this stimulation model was more superior to sham stimulation [22]. Therefore, we adapt this model of rTMS stimulation for individuals with high perceived stress. But for the doses of stimulation, a lower intensity is worth to apply because the targeted subjects may experience mild symptoms compared to depression. Hence, we choose a relatively low stimulation dose (twelve sessions totally, three times per week for four weeks) for patients with high level of perceived stress with reference to similar study by Young In Kim [23]. The sites of stimulation are bilateral dorsolateral prefrontal cortex (DLPFC), which are called F3 (left hemisphere) and F4 (right hemisphere), but using two different types of rTMS intervention: iTBS on F3 and cTBS on F4 [24]. As defined, the standard location of the dorsolateral prefrontal cortex from International 10-20 EEG system is above F3 and F4 [25]. The TMS coil would be moved 6 cm (not 5 cm) anteriorly from the motor cortex where is believed to be the optimal site of stimulation [26]. Combined with motor evoked potentials, single pulse stimulation will be used to determine the resting motor threshold (rMT). The muscle response of abductor pollicis brevis will be observed visually through adjusting the stimulus intensity until the abducted movement occurs in five out of ten times [27]. In addition, all interventions will be carried out by qualified therapists and the responses of the subjects will be recorded after each intervention. And all the subjects will sit on a seat or sofa with extra cervical support for comfortable reasons. All of the interventions will be performed in dose of three times per week for four consecutive weeks.

#### Active rTMS group

The active intervention includes two parts using two different models of TBS on left and right DLPFC. During the first period, the active coil will be placed on the left DLPFC (F3) and the parameters of stimulator will be adjusted properly before treatment. We will apply the iTBS, which contains triplet bursts of 50 Hz repeated at 5 Hz. According to the 2 s on and 8 s off cycle, there are 600 pulses totally (costing approximately 190 s) that will be delivered at 80% of rMT. In the second stimulation part, the stimulator will be located on the right DLPFC (F4) in model of cTBS lasting for 40 s (600 pulses totally).

#### Sham rTMS group

The sham TMS coil (sham Air Film coil, Magstim, Wales, UK) has the same symmetrical mechanical design with active coil and no marks so that it will not be identified from its general shape or appearance. It can also generate periodic noise and tactile sensation similar to those produced by active stimulation, which make it indistinguishable from the active coil by participants. Moreover, the sham coil has a special shield to reduce the magnetic diffusion and stimulate the superficial skin or muscles without generating significant transcranial effect on the brain. The active and sham rTMS conditions will not be differentiable by participants or evaluators.

### Inclusion procedure

Before the beginning of the intervention, individuals who are interested in participation are invited for an appointment to provide more information. If they are willing to participate, informed consent will be signed following the guidance from an independent researcher and the participant will be screened by the inclusion and exclusion criteria. If the subject meets all the inclusion criteria and none of the exclusion criteria, the subject will be included in the study. After inclusion, and before the randomization and allocation, the descriptive data should be collected carefully to get the general information of participants. The basic data are composed of age; gender; race; employment; height; weight; body mass index (BMI); blood pressure; educational level; history of diseases; medication; abuse of smoke, drug, or alcohol; and the habit of exercise. Furthermore, all participants will be assessed by five scales: PSS-14, Depression Anxiety Stress Scale (DASS), Chinese Affective Scale (CAS), Montreal Cognitive Assessment (MoCA), and Test of Nonverbal Intelligence, Third edition (TONI-3). MOCA and TONI-3 are tested as the control factors to ensure there is no significant difference in cognition between the active and sham groups. Biological indicators including sample of serum cortisol will also be collected. Moreover, the anatomy and functions of brain will be scanned by magnetic resonance imaging.

### Measurements

Figure 2 lists the schedule of all study assessments and timeframe in details. The assessments are described briefly below:

### Primary outcome measurement

The primary outcome is the change in perceived stress levels, which is measured by Chinese 14-item perceived stress scales (PSS-14) [28]. The PSS consists of 14 items (7 positive items and 7 negative items) and is a widely used psychological scale to observe the perception of stress in different situation [29]. Participants are required to complete all questions that indicated their perceived stress level within the past several months. Each item is rated on a 5-point scale ranging from 0 (means never) to 4 (means very often), whereas the higher scores indicate higher stress level [30, 31]. Data will be collected at the baseline assessment and the end of intervention (4 weeks after randomization). The mean scores will be calculated for the subsequent statistical analyses.

### Secondary outcome measurements

The secondary outcome measurement includes 2 scales of psychological domains, serum cortisol and neuroimaging data. Data will be collected at the baseline assessment and the end of intervention (4 weeks after randomization). Specifically, the mean scores of the psychological measures, the gray matter density in bilateral DLPFC, the functional connectivity with the DLPFC as seed, and task activation will be calculated as the secondary outcomes. Detailed analyses are stated in the section of “Statistical analysis”.

#### Depression Anxiety Stress Scale (DASS)

The DASS is one of the self-report assessment tools to depression, anxiety, and stress, which is diversely used in different settings. In this trial, the version of DASS 21-items will be used, which consists of three 7-item subscales to describe the emotional statements of subjects over the past week. The score of each item ranges from 0 to 3 in which higher scores mean more severe symptoms. In this version of DASS, “0” indicates “did not apply to me at all” and “3” indicates “applied to me very much, or most of the time” [32].

#### Chinese Affective Scale (CAS)

The 20-item scale CAS offers a brief measurement for trait affect and state of college students and young adults, which was designed for Chinese-speaking individuals. The version of CAS used in this trial contains 10 positive affect and 10 negative affect markers that are rated on 5-point scales (the higher scores, the more severity) and the scale has been demonstrated a good convergent and discriminant validity [33].

#### Biological indicator

The level of serum cortisol in blood samples of both groups will be measured as a biological indicator. The serum cortisol has the ability to adjust individuals’ physiological response to chronic stress while the higher level of perceived stress will indirectly increase the secretion of serum cortisol [34]. Therefore, we choose the serum cortisol as one of the biological indicators. Blood samples are required to be taken when subjects are in quiet condition with a seat for 30 min. Generally, the time of sample collection ranges from 7:30 to 8:30 when participants are in the fasting state. An experienced nurse is responsible for blood sample taking (5 ml) from antecubital vein. Additionally, all subjects are not allowed to drink alcohol 12 h or eating 1 h before the blood draw. The blood sample will also be centrifuged and preserved in − 80 °C within 30 min after sample collection. Finally, serum cortisol will be examined by methods of ELISA [35]. Both blood samples and reagents will be put in room temperature before analysis.

#### Neuroimaging scans

Magnetic resonance imaging (MRI) will be scanned on a 3-T scanner (Siemens, Erlangen, Germany). We acquire multimodal MRI data, including structural MRI, resting state functional MRI, and task functional MRI, to observe the function, anatomy, and connectivity of the brain.

##### Structural MRI

Sagittal plane scanning of MPRAGE T1-weighted sequence will be obtained to observe neural anatomy, such as cerebral white and gray matters. The related parameters are the following: matrix size=256 × 256, number of contiguous slices=192, voxel resolution=1 × 1 × 1 mm, field of view=256 × 256mm, repetition time=2530ms, echo time=2.51ms, and flip angle=7°.

##### Resting state fMRI

The image of brain in axial plane will be scanned using the gradient-echo echo-planar imaging sequence to explore the intrinsic brain activities and function connectivity at the state of rest. One hundred and eighty time points will be scanned during rest. The instructions (“relax, don’t fall asleep, and stare at the cross label on the screen”) will be given to the subject during the scanning period.

##### Task fMRI

To capture the brain functional activations during tasks, the gradient-echo echo-planar imaging sequence will be used to scan, while the participants are required to complete an Emotional Stroop Task, which may cost approximately 10 min. The task [36] is a two-run modified emotion-word Stroop. Each run consists of five randomized blocks. Each block comprises of 12 trials and each trial lasts for 3s. There is a 12-s interval between each block. In each trial, a target emotional word (positive, negative, neutral, congruent color or incongruent color) printed in a color (red, blue, yellow or green) is shown above the fixation cross, while a color word printed in white is shown below the fixation cross. The participants are required to decide whether the meaning of the color word below matched the color of the target emotional word above.

### Safety assessments

During the whole period of intervention, any adverse events (i.e., cannot tolerate MRI procedures, headache, or any discomfort triggered by rTMS stimulation, decreased sleep quality, and seizures) will be recorded per session after intervention immediately. In addition, spontaneous reporting is also requested if there are any abnormalities or adverse events happened during the trial. To ensure safety, the TMS operator should be familiar with the trial scheme and procedure, and all procedures that are related to safety and ethical considerations will follow the guidelines of TMS application strictly [11]. Whenever adverse event happens during the interventional period, the participants will get related medical care accordingly. In addition, all details will be recorded and reported to primary investigator and the Ethic Committee to decide whether the subject is appropriate to continue the trial. All adverse events collected will be truthfully reported in future trial publications and the incidence of adverse events will also be considered into analysis.

### Protocol amendments

This trial will be carried out according to the study protocol version 1. Any modifications of the protocol will be formally amended and submitted to the Ethics Committee of the Affiliated Hospital of Fujian University of Traditional Chinese Medicine.

### Sample size calculation

We aim to investigate the efficacy of the technique of rTMS for improving cognitive emotional control in high-level perceived stress individuals. Since there are no studies of the rTMS efficacy on perceived stress, we have referred to related literature that investigated the effect of rTMS on depression [37], and the sample size estimation was based on the improvement of Hamilton Depression Rating scale (HDRS-17) scores. According to a previous study [37], the mean and its SD of HDRS-17 scores after a 4-month active rTMS intervention (10 subjects included) for treatment-resistant depression were 7.70 and 4.34, respectively, whereas they were 12.29 and 4.50 in the sham rTMS group (7 subjects included), respectively, resulting an effect size (Cohen’s *D*) of 1.038. Referencing from these, a sample size of 32 participants was calculated to sufficiently detect the target effect size (1.04) with a type I error of 5% (*α* = 0.05) and 80% power (*β* = 0.20) by Gpower V.3.1.9.2 software. However, to overcome a dropout rate of 20%, we increased the number of enrollments and finally 40 participants will be allocated randomly into the active and sham stimulation groups with 20 participants in each group for the completion of this trial.

### Randomization and allocation concealment

After the baseline evaluation, each eligible participant will be randomly allocated into either active group or control group with a ratio of 1:1, according to the principle of random permuted blocks. The randomized number and sequence will be generated through SPSS Version 25 by an independent statistician from the Center of Evidence Based Medicine, who will not take part in assessments and execution in this trial. And the therapist and all eligible participants will be informed of the results of group allocation by an independent clinician via telephone.

### Blinding

This trial is a single blind study: both the researchers (outcome assessors and statistical analysts) and participants are blinded to the group assignment and the blindness will never be broken prior to the completion of study unless adverse events happen. But for clinicians or therapists administering at the intervention protocol, it is impossible for them to be blinded. Meanwhile, the randomized sequence of allocation will be replaced by some unrelated codes, such as A and B. In this study, code A means active group and code B means control group. Each parameter of randomization will be kept in a special sealed opaque envelope.

### Data collection and management

At first, the screeners will ensure the inclusion and exclusion criteria and collect basic characteristic data before the randomization and allocation. The trained outcome evaluators, who are blinded to the assignments and not engaged in the intervention, play an important role in measurements of primary and secondary outcomes. Then, the data of all participants will be entered into the designed Case Report Forms (CRF). Besides, each CRF will be checked twice to ensure the accuracy and completion of data collection throughout the study. Meanwhile, all related documents will be coded by specific identification codes and kept in locked boxes to protect participants’ privacy and data security. Furthermore, the MRI scans and biological indicators will be stored on a locked computer with a password that only the primary researcher knows. According to the rules of medical files preservation and principle of Good Clinical Practice (GCP), these materials will be preserved for 5 years after completion of this trial.

In addition, this trial will be monitored for safety control by a physical therapist and two clinical physicians who have no direct involvement in the study. They will be responsible for ensuring the training of standard operation procedure (SOP), monitoring study progress, reviewing all adverse events on a weekly basis, and discontinuing the study if the data raises sufficient concern (e.g., interventions cause treatment site discomfort and headache). All adverse events will be reported to DSMB and to the Ethic Committee of Affiliated Rehabilitation Hospital of Fujian University of Traditional Chinese Medicine by the study evaluators.

### Statistical analysis

All statistical analyses of non-imaging data will be conducted using SPSS Version 25 by an independent statistician who is blinded to the labeling of active and sham groups. The statistical significance level of test is established at 0.05, two-tailed. In the process of data analysis, continuous variables will be represented using mean, standard deviation, maximum or minimum according to the statistical distribution, and the categorical data will be presented by percentage. To verify the efficacy of our intervention, comparison of all outcomes which are categorized as continuous variable and shown in mean scores will be included into analysis by independent sample t test or non-parametric test (Mann-Whitney *U* test). Moreover, both analysis within group and between groups after 4-week intervention will be carried to observe the difference of these two interventional methods. If necessary, logistic regression, general linear correlation or regression will be taken into consideration between different types of outcomes. Similarly, according to the type and distribution of variables, the comparison of basic data between the active group and sham rTMS group will be performed using Student’s *t* test, chi-squared test, or nonparametric test. Furthermore, the participants who drop out during the intervention period will not be excluded due to the principle of Intention to treat (ITT) and the missing data will be replaced by last observation carried forward rules.

The hypothesis of this trial is that the active stimulation group shows greater changes than the sham stimulation group. Therefore, the main analysis is the comparison of changes between groups, measured at baseline and at the end of intervention. If significant differences are observed after intervention and no significant difference is observed at the baseline, we may conclude the efficacy of rTMS to individuals with high level of perceived stress.

In addition, the left and right side of DLPFC will be set as the seed regions for the analysis of the structural and functional MRI data to examine the longitudinal neural changes and to explore the relationship between the brain plasticity and perceived stress caused by the stimulation. Specifically, the gray matter density in bilateral DLPFC, the functional connectivity with the DLPFC as seed, and task activation will be calculated using statistical parametric mapping software (SPM12, Welcome Department of Cognitive Neurology, London, UK, http://www.fil.ion.ucl.ac.uk/spm/). A two-factor repeated measures ANOVA (group factor for active and sham groups, and two longitudinal measures at baseline and after intervention) will be conducted on the gray matter density, functional connectivity, and the task activation separately. Further correlational analyses will be performed between the imaging and the psychological and biological measures to explore the relationship between brain and perceived stress.

### Dissemination of research results

Before obtaining participants’ informed consent, they will be inquired whether their basic information could be used in our future publications. Only if agree, this participant will be included into our trial and every one included will be requested to sign their name in our informed consent. Moreover, to guarantee their right to know their own results, each participant will receive a full result report of our test. After completion of all intervention, we plan to manage and publish our trial results in some peer-reviewed journals or related conferences. For future trial publications, all researchers and other colleagues who participated in this study will be co-authors of the study based on their individual contributions.

## Discussion

Perceived stress is a big problem among people with all age groups, which may lead to some negative effect on personal health. But the suitable treatment for perceived stress is still in practice. RTMS has become a popular intervention for psychiatric disorders because of its advantages: non-invasiveness, painless, and well tolerability [8, 38]. As a new pattern of rTMS intervention, TBS has been successfully established to trigger the changes of cerebral cortex excitability [18, 24]. Compared to the conventional rTMS protocol, TBS has stronger and longer lasting effects with lower frequency of stimulation and shorter time [39]. Moreover, it has been demonstrated that intermittent TBS has better effects on increasing the cortical excitability than high frequency of rTMS and continuous TBS is superior to the low frequency rTMS in reduction of motor cortex excitability [18, 40]. Following the recommended stimulation protocol from recent TMS guideline for stress-related disorder (i.e., depression), iTBS and cTBS will be applied in left and right DLFPFC respectively to adjust the cortical excitability [12]. Moreover, the evidence shows that 600 pulses of iTBS and cTBS can generate the longest lasting effects (60 min and 50 min respectively) than any other frequency of TBS stimulation [41]. Therefore, 600 pulses are set as the frequency in this trial.

Most of TMS-related studies use the 5-cm rules to localize the DLPFC (above Brodman areas 9 and 46) by evoking a response the abductor pollicis brevis muscle in contralateral hand and then moving the coil 5 cm anteriorly. However, several studies with neuroimaging navigation systems pointed that the 5-cm rule would not always target the DLPFC exactly in all subjects due to the individual variability (i.e., head size), and the location of DLPFC is more anteriorly than 5cm in the majority of individuals [12, 42, 43]. Therefore, we decide to move 6 cm anteriorly rather than 5 cm following an evolution of TMS protocols over several clinical trials [26, 44].

Cortisol has the ability to adjust individuals’ physiological response to chronic stress and the stress-related elevation of cortisol has a negative effect to the metabolism and synaptic density of hippocampus and prefrontal cortex in brain [34]. Previous clinical reviews suggest that in the stress response cycle in blood, high level of perceived stress will increase the amount of release of Adrenocorticotropic Hormone (ACTH), which is associated with the HPA axis. Then the adrenal cortex will be activated by ACTH to produce more cortisol [7, 45]. Therefore, the indexes of serum cortisol will be measured from blood sample in our trial.

The potential limitation of this trial is that it is not a double-blinded controlled trial. Although we attempt to make everyone blind, it is not always feasible in all non-pharmacological studies [46]. It is impossible to blind the therapists or clinicians who are responsible for replacing the sham coil for participants in sham-controlled group. However, the outcome evaluators and statisticians will be masked throughout the whole trial to eliminate any possible bias. Besides, we have set a sham stimulation group to eliminate the placebo effect of rTMS intervention. Secondly, the changes of perceived stress, biological or neural measured observed right after intervention may be transient [47]. To make it a realistic therapeutic intervention, additional follow-up evaluations after a period of time are needed. Thirdly, the small sample size of participants included may limit the generalization of the findings.

To conclude, the protocol of this randomized clinical trial will investigate whether 12 sessions of TBS stimulations affect the perceived stress and help to explain the possible mechanisms underlying the stimulation. Positive results of our trial may provide a new choice and evidence for the treatment application of individuals with high level of perceived stress.

## Study status

This is version 2.0 of the protocol, dated 23 November 2019. Recruitment began on 7 June 2020. Recruitment is predicted to continue until October 2021.And at the time of submission, participant recruitment is still ongoing.

## Supplementary Information

**Additional file 1.** SPIRIT checklist.

## Data Availability

After the completion of the trial, only researchers or teams with ethical approval will have access to the final datasets. The datasets analyzed during the current study will be available from the corresponding author on reasonable request.

## Abbreviations

ACTH: Adrenocorticotropic Hormone
BMI: Body mass index
CAS: Chinese Affective Scale
CRF: Case report form
cTBS: Continuous theta burst stimulation
DASS: Depression, anxiety and stress scale
DLPFC: Dorsal lateral prefrontal cortex
ELISA: Enzyme-linked immunosorbent assay
FA: Flip angle
FDA: Food and Drug Administration
FJTCM: Fujian University of Traditional Chinese Medicine
FOV: Field of view
GCP: Good Clinical Practice
HPA: Hypothalamic-pituitary-adrenal
iTBS: Intermittent theta burst stimulation
ITT: Intention to treat
MOCA: Montreal Cognitive Assessment
MRI: Magnetic resonance imaging
PSS: Perceived stress scale
rMT: Resting motor threshold
TE: Echo time
TR: Repetition time
